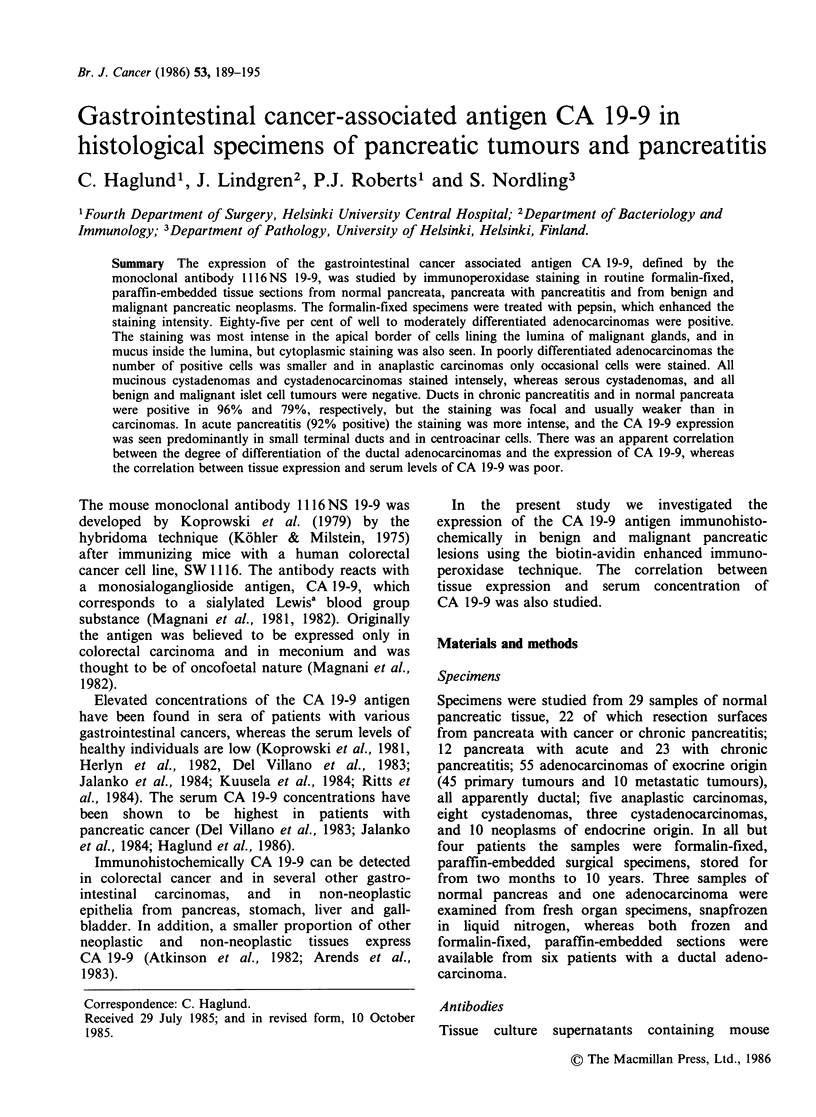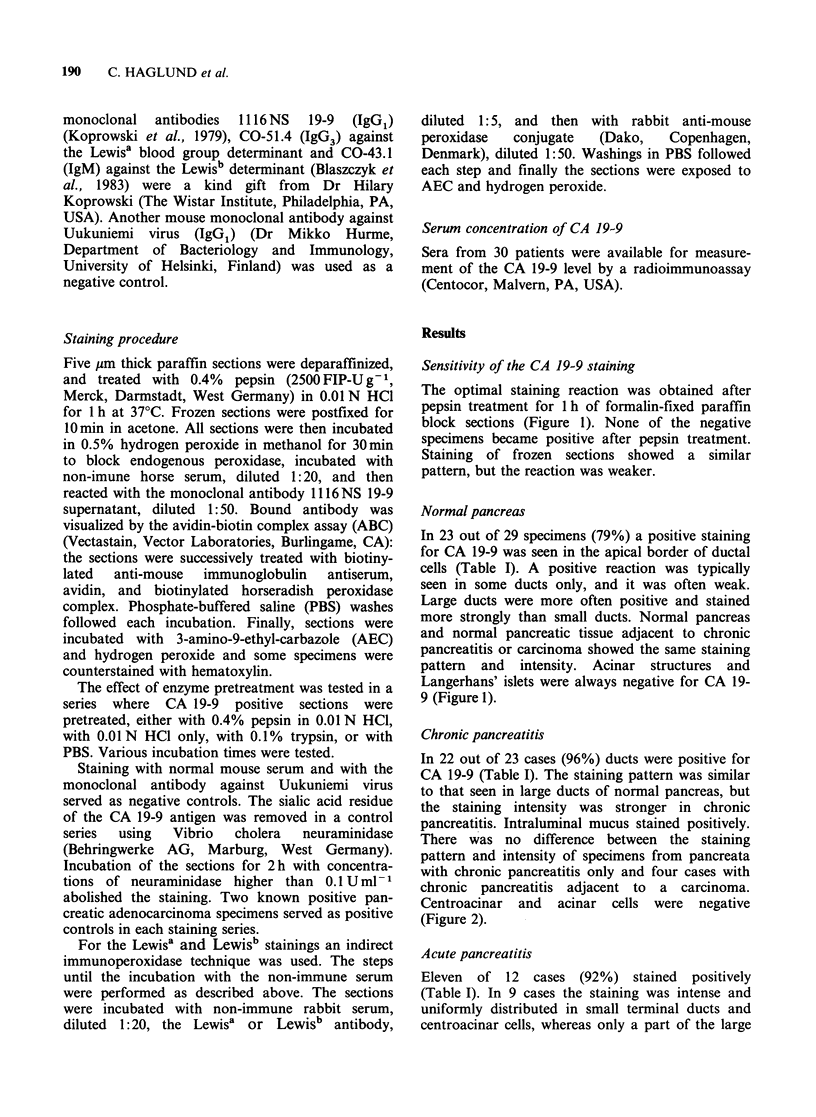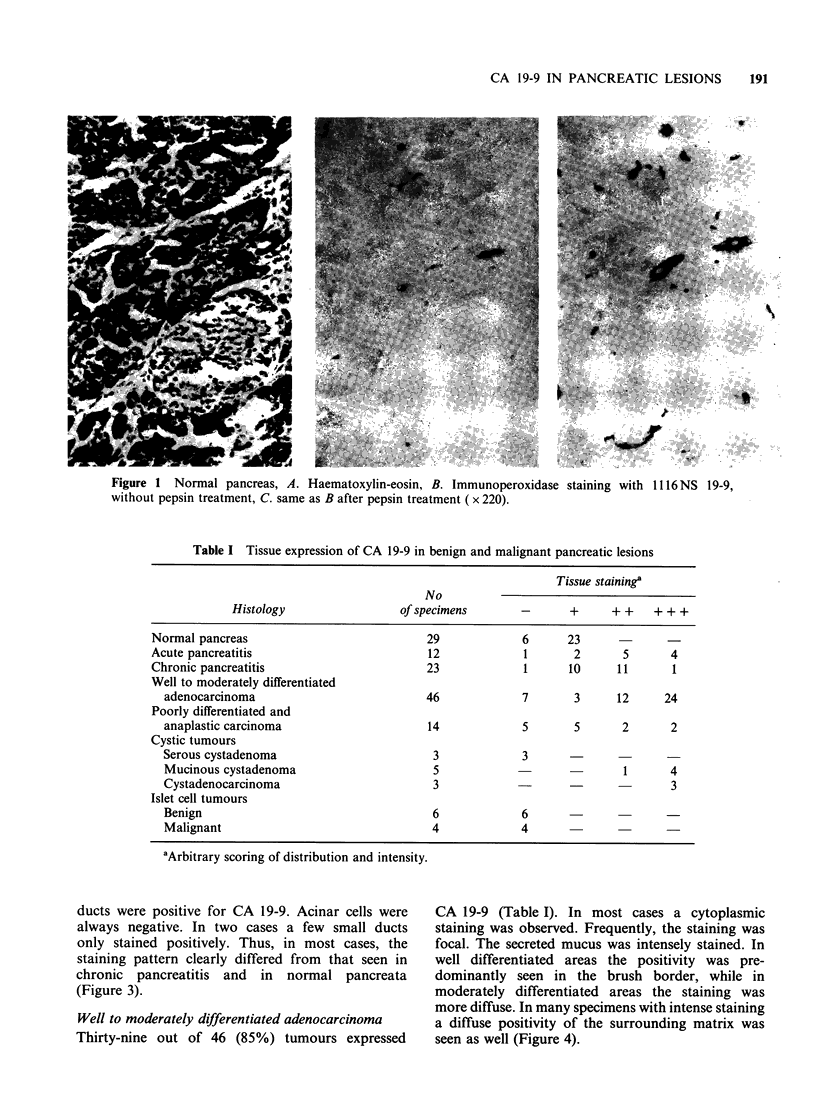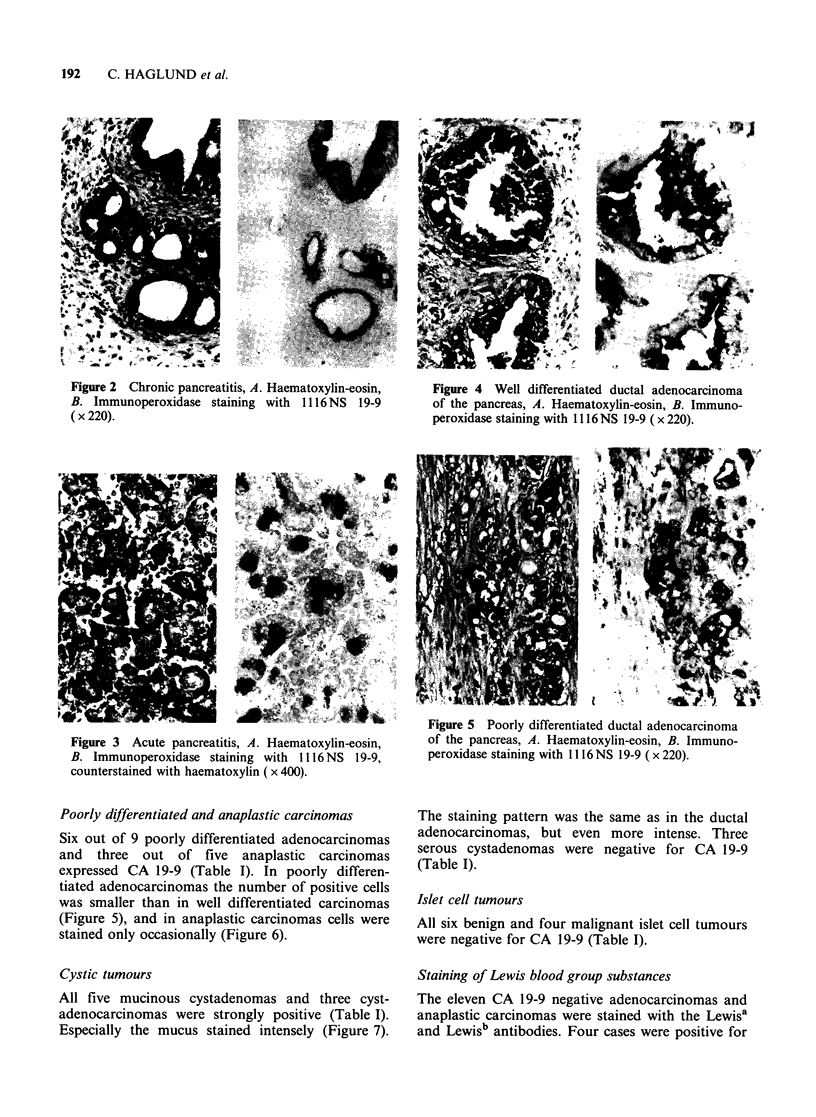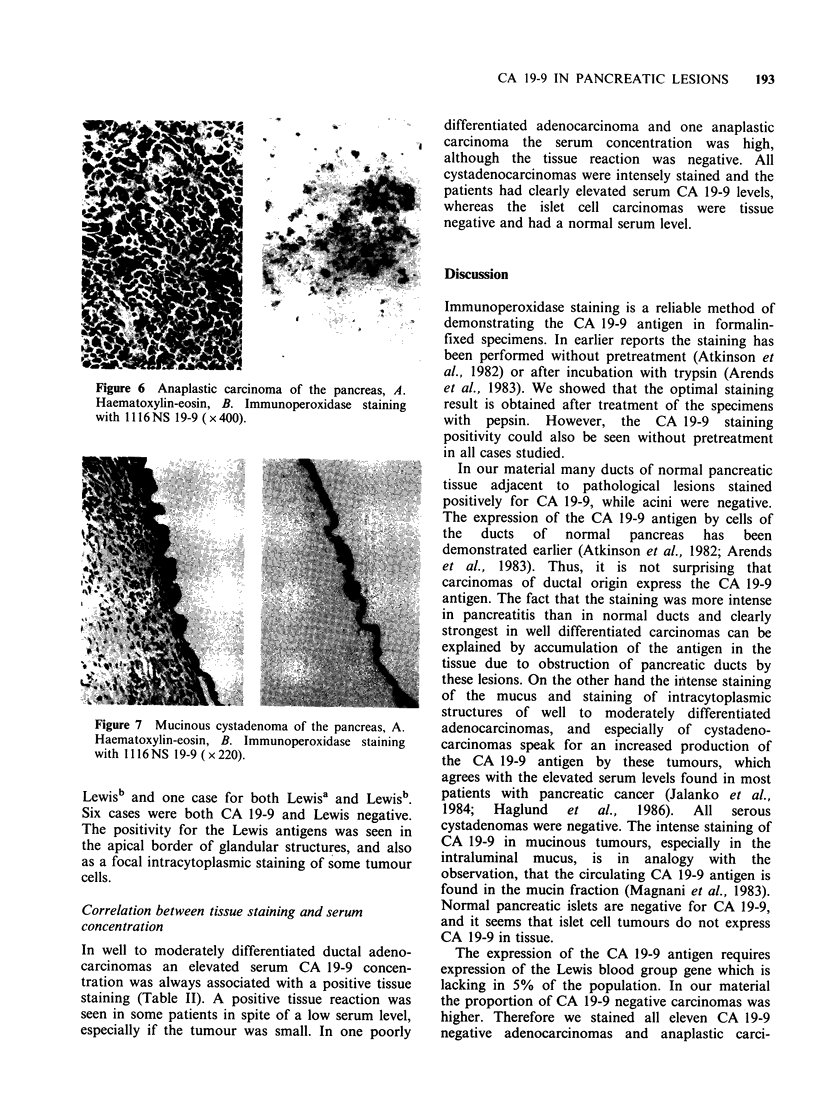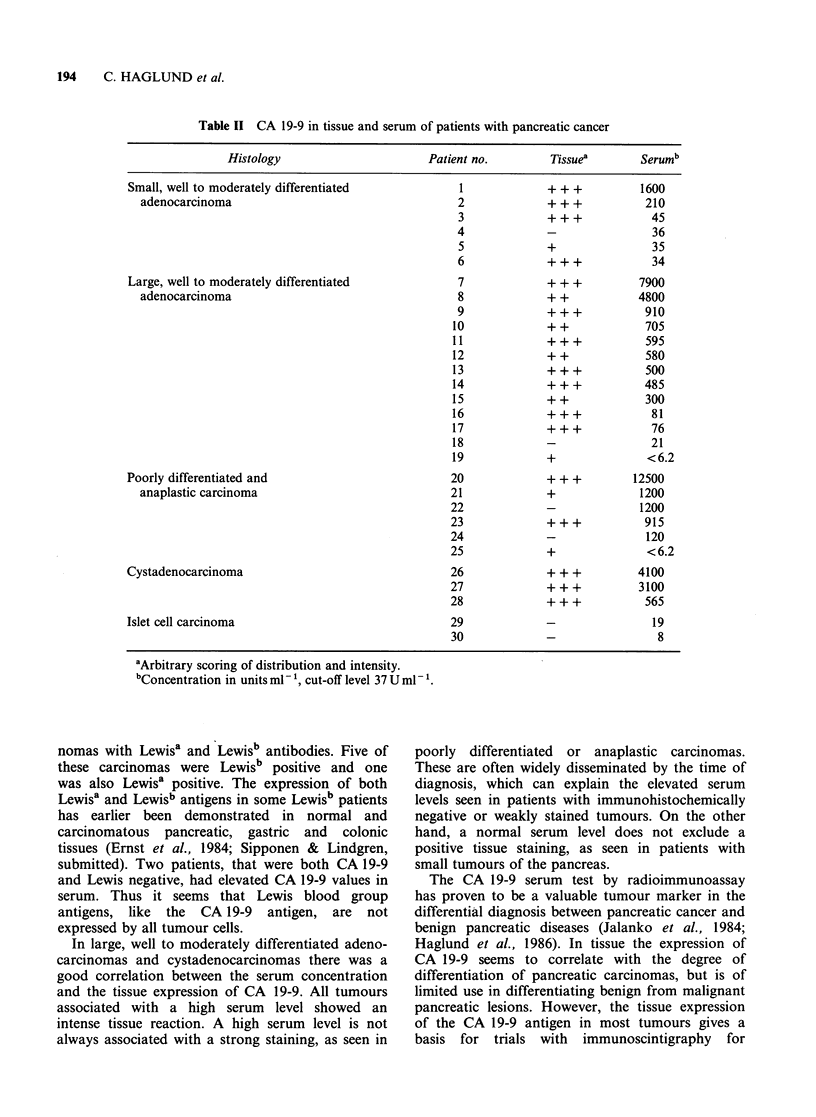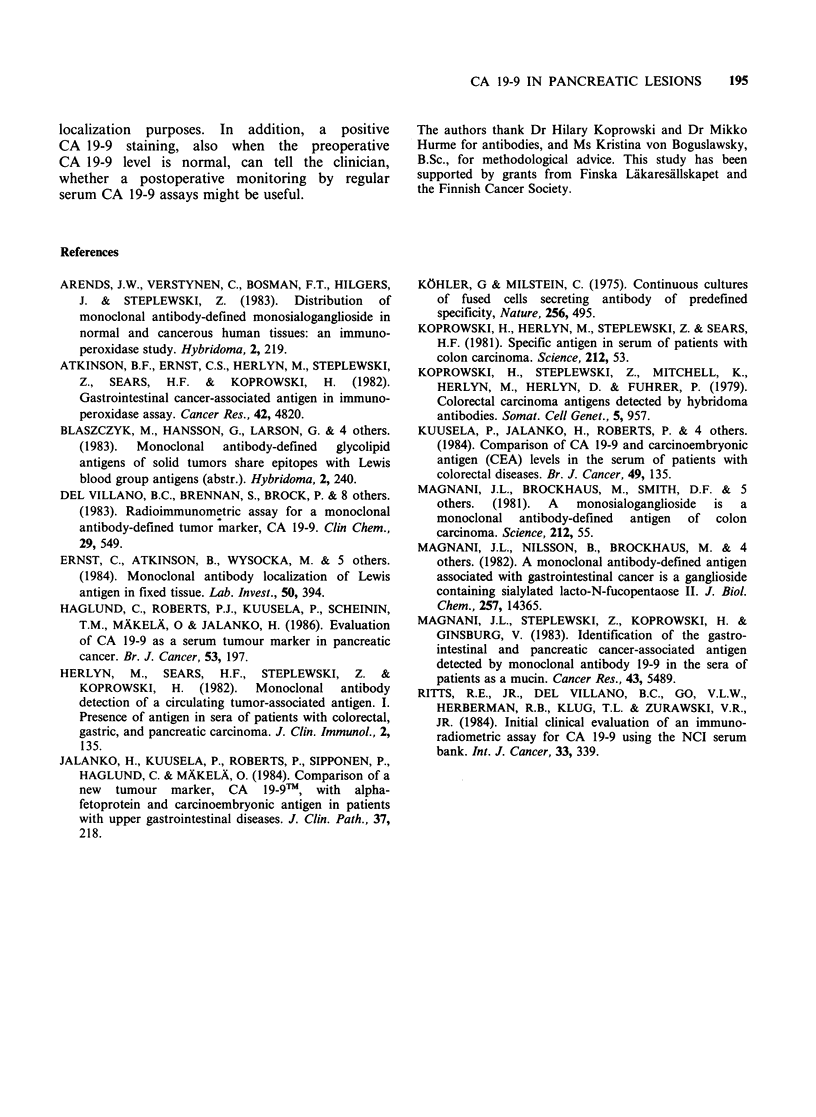# Gastrointestinal cancer-associated antigen CA 19-9 in histological specimens of pancreatic tumours and pancreatitis.

**DOI:** 10.1038/bjc.1986.34

**Published:** 1986-02

**Authors:** C. Haglund, J. Lindgren, P. J. Roberts, S. Nordling

## Abstract

**Images:**


Br. J. Cancer (1986) 53, 189–195

### Gastrointestinal cancer-associated antigen CA 19-9 in histological specimens of pancreatic tumours and pancreatitis

C. Haglund[1], J. Lindgren[2], P.J. Roberts[1] and S. Nordling[3]

[1]*Fourth Department of Surgery, Helsinki University Central Hospital;* [2]*Department of Bacteriology and Immunology;* [3]*Department of Pathology, University of Helsinki, Helsinki, Finland.*

**Summary** The expression of the gastrointestinal cancer associated antigen CA 19-9, defined by the monoclonal antibody 1116NS 19-9, was studied by immunoperoxidase staining in routine formalin-fixed, paraffin-embedded tissue sections from normal pancreata, pancreata with pancreatitis and from benign and malignant pancreatic neoplasms. The formalin-fixed specimens were treated with pepsin, which enhanced the staining intensity. Eighty-five per cent of well to moderately differentiated adenocarcinomas were positive. The staining was most intense in the apical border of cells lining the lumina of malignant glands, and in mucus inside the lumina, but cytoplasmic staining was also seen. In poorly differentiated adenocarcinomas the number of positive cells was smaller and in anaplastic carcinomas only occasional cells were stained. All mucinous cystadenomas and cystadenocarcinomas stained intensely, whereas serous cystadenomas, and all benign and malignant islet cell tumours were negative. Ducts in chronic pancreatitis and in normal pancreata were positive in 96% and 79%, respectively, but the staining was focal and usually weaker than in carcinomas. In acute pancreatitis (92% positive) the staining was more intense, and the CA 19-9 expression was seen predominantly in small terminal ducts and in centroacinar cells. There was an apparent correlation between the degree of differentiation of the ductal adenocarcinomas and the expression of CA 19-9, whereas the correlation between tissue expression and serum levels of CA 19-9 was poor.

The mouse monoclonal antibody 1116NS 19-9 was developed by Koprowski *et al.* (1979) by the hybridoma technique (Köhler & Milstein, 1975) after immunizing mice with a human colorectal cancer cell line, SW1116. The antibody reacts with a monosialoganglioside antigen, CA 19-9, which corresponds to a sialylated Lewis[a] blood group substance (Magnani *et al.*, 1981, 1982). Originally the antigen was believed to be expressed only in colorectal carcinoma and in meconium and was thought to be of oncofoetal nature (Magnani *et al.*, 1982).

Elevated concentrations of the CA 19-9 antigen have been found in sera of patients with various gastrointestinal cancers, whereas the serum levels of healthy individuals are low (Koprowski *et al.*, 1981, Herlyn *et al.*, 1982, Del Villano *et al.*, 1983; Jalanko *et al.*, 1984; Kuusela *et al.*, 1984; Ritts *et al.*, 1984). The serum CA 19-9 concentrations have been shown to be highest in patients with pancreatic cancer (Del Villano *et al.*, 1983; Jalanko *et al.*, 1984; Haglund *et al.*, 1986).

Immunohistochemically CA 19-9 can be detected in colorectal cancer and in several other gastrointestinal carcinomas, and in non-neoplastic epithelia from pancreas, stomach, liver and gallbladder. In addition, a smaller proportion of other neoplastic and non-neoplastic tissues express CA 19-9 (Atkinson *et al.*, 1982; Arends *et al.*, 1983).

Correspondence: C. Haglund.
Received 29 July 1985; and in revised form, 10 October 1985.

In the present study we investigated the expression of the CA 19-9 antigen immunohistochemically in benign and malignant pancreatic lesions using the biotin-avidin enhanced immunoperoxidase technique. The correlation between tissue expression and serum concentration of CA 19-9 was also studied.

### Materials and methods

### *Specimens*

Specimens were studied from 29 samples of normal pancreatic tissue, 22 of which resection surfaces from pancreata with cancer or chronic pancreatitis; 12 pancreata with acute and 23 with chronic pancreatitis; 55 adenocarcinomas of exocrine origin (45 primary tumours and 10 metastatic tumours), all apparently ductal; five anaplastic carcinomas, eight cystadenomas, three cystadenocarcinomas, and 10 neoplasms of endocrine origin. In all but four patients the samples were formalin-fixed, paraffin-embedded surgical specimens, stored for from two months to 10 years. Three samples of normal pancreas and one adenocarcinoma were examined from fresh organ specimens, snapfrozen in liquid nitrogen, whereas both frozen and formalin-fixed, paraffin-embedded sections were available from six patients with a ductal adenocarcinoma.

### *Antibodies*

Tissue culture supernatants containing mouse

© The Macmillan Press, Ltd., 1986

monoclonal antibodies 1116NS 19-9 ($IgG_1$) (Koprowski *et al.*, 1979), CO-51.4 ($IgG_3$) against the Lewis[a] blood group determinant and CO-43.1 (IgM) against the Lewis[b] determinant (Blaszczyk *et al.*, 1983) were a kind gift from Dr Hilary Koprowski (The Wistar Institute, Philadelphia, PA, USA). Another mouse monoclonal antibody against Uukuniemi virus ($IgG_1$) (Dr Mikko Hurme, Department of Bacteriology and Immunology, University of Helsinki, Finland) was used as a negative control.

*Staining procedure*

Five μm thick paraffin sections were deparaffinized, and treated with 0.4% pepsin (2500 FIP-U $g^{-1}$, Merck, Darmstadt, West Germany) in 0.01 N HCl for 1 h at 37°C. Frozen sections were postfixed for 10 min in acetone. All sections were then incubated in 0.5% hydrogen peroxide in methanol for 30 min to block endogenous peroxidase, incubated with non-imune horse serum, diluted 1:20, and then reacted with the monoclonal antibody 1116NS 19-9 supernatant, diluted 1:50. Bound antibody was visualized by the avidin-biotin complex assay (ABC) (Vectastain, Vector Laboratories, Burlingame, CA): the sections were successively treated with biotinylated anti-mouse immunoglobulin antiserum, avidin, and biotinylated horseradish peroxidase complex. Phosphate-buffered saline (PBS) washes followed each incubation. Finally, sections were incubated with 3-amino-9-ethyl-carbazole (AEC) and hydrogen peroxide and some specimens were counterstained with hematoxylin.

The effect of enzyme pretreatment was tested in a series where CA 19-9 positive sections were pretreated, either with 0.4% pepsin in 0.01 N HCl, with 0.01 N HCl only, with 0.1% trypsin, or with PBS. Various incubation times were tested.

Staining with normal mouse serum and with the monoclonal antibody against Uukuniemi virus served as negative controls. The sialic acid residue of the CA 19-9 antigen was removed in a control series using Vibrio cholera neuraminidase (Behringwerke AG, Marburg, West Germany). Incubation of the sections for 2 h with concentrations of neuraminidase higher than 0.1 U $ml^{-1}$ abolished the staining. Two known positive pancreatic adenocarcinoma specimens served as positive controls in each staining series.

For the Lewis[a] and Lewis[b] stainings an indirect immunoperoxidase technique was used. The steps until the incubation with the non-immune serum were performed as described above. The sections were incubated with non-immune rabbit serum, diluted 1:20, the Lewis[a] or Lewis[b] antibody, diluted 1:5, and then with rabbit anti-mouse peroxidase conjugate (Dako, Copenhagen, Denmark), diluted 1:50. Washings in PBS followed each step and finally the sections were exposed to AEC and hydrogen peroxide.

*Serum concentration of CA 19-9*

Sera from 30 patients were available for measurement of the CA 19-9 level by a radioimmunoassay (Centocor, Malvern, PA, USA).

### Results

*Sensitivity of the CA 19-9 staining*

The optimal staining reaction was obtained after pepsin treatment for 1 h of formalin-fixed paraffin block sections (Figure 1). None of the negative specimens became positive after pepsin treatment. Staining of frozen sections showed a similar pattern, but the reaction was weaker.

*Normal pancreas*

In 23 out of 29 specimens (79%) a positive staining for CA 19-9 was seen in the apical border of ductal cells (Table I). A positive reaction was typically seen in some ducts only, and it was often weak. Large ducts were more often positive and stained more strongly than small ducts. Normal pancreas and normal pancreatic tissue adjacent to chronic pancreatitis or carcinoma showed the same staining pattern and intensity. Acinar structures and Langerhans' islets were always negative for CA 19-9 (Figure 1).

*Chronic pancreatitis*

In 22 out of 23 cases (96%) ducts were positive for CA 19-9 (Table I). The staining pattern was similar to that seen in large ducts of normal pancreas, but the staining intensity was stronger in chronic pancreatitis. Intraluminal mucus stained positively. There was no difference between the staining pattern and intensity of specimens from pancreata with chronic pancreatitis only and four cases with chronic pancreatitis adjacent to a carcinoma. Centroacinar and acinar cells were negative (Figure 2).

*Acute pancreatitis*

Eleven of 12 cases (92%) stained positively (Table I). In 9 cases the staining was intense and uniformly distributed in small terminal ducts and centroacinar cells, whereas only a part of the large

**Figure 1** Normal pancreas, *A*. Haematoxylin-eosin, *B*. Immunoperoxidase staining with 1116NS 19-9, without pepsin treatment, *C*. same as *B* after pepsin treatment ( × 220).

**Table I** Tissue expression of CA 19-9 in benign and malignant pancreatic lesions

| *Histology* | *No of specimens* | *Tissue staining*[a] − | + | ++ | +++ |
|---|---|---|---|---|---|
| Normal pancreas | 29 | 6 | 23 | — | — |
| Acute pancreatitis | 12 | 1 | 2 | 5 | 4 |
| Chronic pancreatitis | 23 | 1 | 10 | 11 | 1 |
| Well to moderately differentiated adenocarcinoma | 46 | 7 | 3 | 12 | 24 |
| Poorly differentiated and anaplastic carcinoma | 14 | 5 | 5 | 2 | 2 |
| Cystic tumours | | | | | |
| Serous cystadenoma | 3 | 3 | — | — | — |
| Mucinous cystadenoma | 5 | — | — | 1 | 4 |
| Cystadenocarcinoma | 3 | — | — | — | 3 |
| Islet cell tumours | | | | | |
| Benign | 6 | 6 | — | — | — |
| Malignant | 4 | 4 | — | — | — |

[a]Arbitrary scoring of distribution and intensity.

ducts were positive for CA 19-9. Acinar cells were always negative. In two cases a few small ducts only stained positively. Thus, in most cases, the staining pattern clearly differed from that seen in chronic pancreatitis and in normal pancreata (Figure 3).

### *Well to moderately differentiated adenocarcinoma*

Thirty-nine out of 46 (85%) tumours expressed CA 19-9 (Table I). In most cases a cytoplasmic staining was observed. Frequently, the staining was focal. The secreted mucus was intensely stained. In well differentiated areas the positivity was predominantly seen in the brush border, while in moderately differentiated areas the staining was more diffuse. In many specimens with intense staining a diffuse positivity of the surrounding matrix was seen as well (Figure 4).

**Figure 2** Chronic pancreatitis, *A*. Haematoxylin-eosin, *B*. Immunoperoxidase staining with 1116NS 19-9 (×220).

**Figure 3** Acute pancreatitis, *A*. Haematoxylin-eosin, *B*. Immunoperoxidase staining with 1116NS 19-9, counterstained with haematoxylin (×400).

**Figure 4** Well differentiated ductal adenocarcinoma of the pancreas, *A*. Haematoxylin-eosin, *B*. Immunoperoxidase staining with 1116NS 19-9 (×220).

**Figure 5** Poorly differentiated ductal adenocarcinoma of the pancreas, *A*. Haematoxylin-eosin, *B*. Immunoperoxidase staining with 1116NS 19-9 (×220).

*Poorly differentiated and anaplastic carcinomas*

Six out of 9 poorly differentiated adenocarcinomas and three out of five anaplastic carcinomas expressed CA 19-9 (Table I). In poorly differentiated adenocarcinomas the number of positive cells was smaller than in well differentiated carcinomas (Figure 5), and in anaplastic carcinomas cells were stained only occasionally (Figure 6).

*Cystic tumours*

All five mucinous cystadenomas and three cystadenocarcinomas were strongly positive (Table I). Especially the mucus stained intensely (Figure 7). The staining pattern was the same as in the ductal adenocarcinomas, but even more intense. Three serous cystadenomas were negative for CA 19-9 (Table I).

*Islet cell tumours*

All six benign and four malignant islet cell tumours were negative for CA 19-9 (Table I).

*Staining of Lewis blood group substances*

The eleven CA 19-9 negative adenocarcinomas and anaplastic carcinomas were stained with the Lewis[a] and Lewis[b] antibodies. Four cases were positive for

**Figure 6** Anaplastic carcinoma of the pancreas, *A*. Haematoxylin-eosin, *B*. Immunoperoxidase staining with 1116NS 19-9 (×400).

**Figure 7** Mucinous cystadenoma of the pancreas, A. Haematoxylin-eosin, *B*. Immunoperoxidase staining with 1116NS 19-9 (×220).

Lewis$^b$ and one case for both Lewis$^a$ and Lewis$^b$. Six cases were both CA 19-9 and Lewis negative. The positivity for the Lewis antigens was seen in the apical border of glandular structures, and also as a focal intracytoplasmic staining of some tumour cells.

*Correlation between tissue staining and serum concentration*

In well to moderately differentiated ductal adenocarcinomas an elevated serum CA 19-9 concentration was always associated with a positive tissue staining (Table II). A positive tissue reaction was seen in some patients in spite of a low serum level, especially if the tumour was small. In one poorly differentiated adenocarcinoma and one anaplastic carcinoma the serum concentration was high, although the tissue reaction was negative. All cystadenocarcinomas were intensely stained and the patients had clearly elevated serum CA 19-9 levels, whereas the islet cell carcinomas were tissue negative and had a normal serum level.

### Discussion

Immunoperoxidase staining is a reliable method of demonstrating the CA 19-9 antigen in formalin-fixed specimens. In earlier reports the staining has been performed without pretreatment (Atkinson *et al.*, 1982) or after incubation with trypsin (Arends *et al.*, 1983). We showed that the optimal staining result is obtained after treatment of the specimens with pepsin. However, the CA 19-9 staining positivity could also be seen without pretreatment in all cases studied.

In our material many ducts of normal pancreatic tissue adjacent to pathological lesions stained positively for CA 19-9, while acini were negative. The expression of the CA 19-9 antigen by cells of the ducts of normal pancreas has been demonstrated earlier (Atkinson *et al.*, 1982; Arends *et al.*, 1983). Thus, it is not surprising that carcinomas of ductal origin express the CA 19-9 antigen. The fact that the staining was more intense in pancreatitis than in normal ducts and clearly strongest in well differentiated carcinomas can be explained by accumulation of the antigen in the tissue due to obstruction of pancreatic ducts by these lesions. On the other hand the intense staining of the mucus and staining of intracytoplasmic structures of well to moderately differentiated adenocarcinomas, and especially of cystadenocarcinomas speak for an increased production of the CA 19-9 antigen by these tumours, which agrees with the elevated serum levels found in most patients with pancreatic cancer (Jalanko *et al.*, 1984; Haglund *et al.*, 1986). All serous cystadenomas were negative. The intense staining of CA 19-9 in mucinous tumours, especially in the intraluminal mucus, is in analogy with the observation, that the circulating CA 19-9 antigen is found in the mucin fraction (Magnani *et al.*, 1983). Normal pancreatic islets are negative for CA 19-9, and it seems that islet cell tumours do not express CA 19-9 in tissue.

The expression of the CA 19-9 antigen requires expression of the Lewis blood group gene which is lacking in 5% of the population. In our material the proportion of CA 19-9 negative carcinomas was higher. Therefore we stained all eleven CA 19-9 negative adenocarcinomas and anaplastic carci-

**Table II** CA 19-9 in tissue and serum of patients with pancreatic cancer

| *Histology* | *Patient no.* | *Tissue*[a] | *Serum*[b] |
|---|---|---|---|
| Small, well to moderately differentiated adenocarcinoma | 1 | + + + | 1600 |
| | 2 | + + + | 210 |
| | 3 | + + + | 45 |
| | 4 | − | 36 |
| | 5 | + | 35 |
| | 6 | + + + | 34 |
| Large, well to moderately differentiated adenocarcinoma | 7 | + + + | 7900 |
| | 8 | + + | 4800 |
| | 9 | + + + | 910 |
| | 10 | + + | 705 |
| | 11 | + + + | 595 |
| | 12 | + + | 580 |
| | 13 | + + + | 500 |
| | 14 | + + + | 485 |
| | 15 | + + | 300 |
| | 16 | + + + | 81 |
| | 17 | + + + | 76 |
| | 18 | − | 21 |
| | 19 | + | <6.2 |
| Poorly differentiated and anaplastic carcinoma | 20 | + + + | 12500 |
| | 21 | + | 1200 |
| | 22 | − | 1200 |
| | 23 | + + + | 915 |
| | 24 | − | 120 |
| | 25 | + | <6.2 |
| Cystadenocarcinoma | 26 | + + + | 4100 |
| | 27 | + + + | 3100 |
| | 28 | + + + | 565 |
| Islet cell carcinoma | 29 | − | 19 |
| | 30 | − | 8 |

[a]Arbitrary scoring of distribution and intensity.
[b]Concentration in units $ml^{-1}$, cut-off level 37 U $ml^{-1}$.

nomas with Lewis[a] and Lewis[b] antibodies. Five of these carcinomas were Lewis[b] positive and one was also Lewis[a] positive. The expression of both Lewis[a] and Lewis[b] antigens in some Lewis[b] patients has earlier been demonstrated in normal and carcinomatous pancreatic, gastric and colonic tissues (Ernst *et al.*, 1984; Sipponen & Lindgren, submitted). Two patients, that were both CA 19-9 and Lewis negative, had elevated CA 19-9 values in serum. Thus it seems that Lewis blood group antigens, like the CA 19-9 antigen, are not expressed by all tumour cells.

In large, well to moderately differentiated adenocarcinomas and cystadenocarcinomas there was a good correlation between the serum concentration and the tissue expression of CA 19-9. All tumours associated with a high serum level showed an intense tissue reaction. A high serum level is not always associated with a strong staining, as seen in poorly differentiated or anaplastic carcinomas. These are often widely disseminated by the time of diagnosis, which can explain the elevated serum levels seen in patients with immunohistochemically negative or weakly stained tumours. On the other hand, a normal serum level does not exclude a positive tissue staining, as seen in patients with small tumours of the pancreas.

The CA 19-9 serum test by radioimmunoassay has proven to be a valuable tumour marker in the differential diagnosis between pancreatic cancer and benign pancreatic diseases (Jalanko *et al.*, 1984; Haglund *et al.*, 1986). In tissue the expression of CA 19-9 seems to correlate with the degree of differentiation of pancreatic carcinomas, but is of limited use in differentiating benign from malignant pancreatic lesions. However, the tissue expression of the CA 19-9 antigen in most tumours gives a basis for trials with immunoscintigraphy for localization purposes. In addition, a positive CA 19-9 staining, also when the preoperative CA 19-9 level is normal, can tell the clinician, whether a postoperative monitoring by regular serum CA 19-9 assays might be useful.

The authors thank Dr Hilary Koprowski and Dr Mikko Hurme for antibodies, and Ms Kristina von Boguslawsky, B.Sc., for methodological advice. This study has been supported by grants from Finska Läkaresällskapet and the Finnish Cancer Society.

### References

ARENDS, J.W., VERSTYNEN, C., BOSMAN, F.T., HILGERS, J. & STEPLEWSKI, Z. (1983). Distribution of monoclonal antibody-defined monosialoganglioside in normal and cancerous human tissues: an immunoperoxidase study. *Hybridoma,* **2,** 219.

ATKINSON, B.F., ERNST, C.S., HERLYN, M., STEPLEWSKI, Z., SEARS, H.F. & KOPROWSKI, H. (1982). Gastrointestinal cancer-associated antigen in immunoperoxidase assay. *Cancer Res.,* **42,** 4820.

BLASZCZYK, M., HANSSON, G., LARSON, G. & 4 others. (1983). Monoclonal antibody-defined glycolipid antigens of solid tumors share epitopes with Lewis blood group antigens (abstr.). *Hybridoma,* **2,** 240.

DEL VILLANO, B.C., BRENNAN, S., BROCK, P. & 8 others. (1983). Radioimmunometric assay for a monoclonal antibody-defined tumor marker, CA 19-9. *Clin Chem.,* **29,** 549.

ERNST, C., ATKINSON, B., WYSOCKA, M. & 5 others. (1984). Monoclonal antibody localization of Lewis antigen in fixed tissue. *Lab. Invest.,* **50,** 394.

HAGLUND, C., ROBERTS, P.J., KUUSELA, P., SCHEININ, T.M., MÄKELÄ, O & JALANKO, H. (1986). Evaluation of CA 19-9 as a serum tumour marker in pancreatic cancer. *Br. J. Cancer,* **53,** 197.

HERLYN, M., SEARS, H.F., STEPLEWSKI, Z. & KOPROWSKI, H. (1982). Monoclonal antibody detection of a circulating tumor-associated antigen. I. Presence of antigen in sera of patients with colorectal, gastric, and pancreatic carcinoma. *J. Clin. Immunol.,* **2,** 135.

JALANKO, H., KUUSELA, P., ROBERTS, P., SIPPONEN, P., HAGLUND, C. & MÄKELÄ, O. (1984). Comparison of a new tumour marker, CA 19-9™, with alphafetoprotein and carcinoembryonic antigen in patients with upper gastrointestinal diseases. *J. Clin. Path.,* **37,** 218.

KÖHLER, G & MILSTEIN, C. (1975). Continuous cultures of fused cells secreting antibody of predefined specificity, *Nature,* **256,** 495.

KOPROWSKI, H., HERLYN, M., STEPLEWSKI, Z. & SEARS, H.F. (1981). Specific antigen in serum of patients with colon carcinoma. *Science,* **212,** 53.

KOPROWSKI, H., STEPLEWSKI, Z., MITCHELL, K., HERLYN, M., HERLYN, D. & FUHRER, P. (1979). Colorectal carcinoma antigens detected by hybridoma antibodies. *Somat. Cell Genet.,* **5,** 957.

KUUSELA, P., JALANKO, H., ROBERTS, P. & 4 others. (1984). Comparison of CA 19-9 and carcinoembryonic antigen (CEA) levels in the serum of patients with colorectal diseases. *Br. J. Cancer,* **49,** 135.

MAGNANI, J.L., BROCKHAUS, M., SMITH, D.F. & 5 others. (1981). A monosialoganglioside is a monoclonal antibody-defined antigen of colon carcinoma. *Science,* **212,** 55.

MAGNANI, J.L., NILSSON, B., BROCKHAUS, M. & 4 others. (1982). A monoclonal antibody-defined antigen associated with gastrointestinal cancer is a ganglioside containing sialylated lacto-N-fucopentaose II. *J. Biol. Chem.,* **257,** 14365.

MAGNANI, J.L., STEPLEWSKI, Z., KOPROWSKI, H. & GINSBURG, V. (1983). Identification of the gastrointestinal and pancreatic cancer-associated antigen detected by monoclonal antibody 19-9 in the sera of patients as a mucin. *Cancer Res.,* **43,** 5489.

RITTS, R.E., JR., DEL VILLANO, B.C., GO, V.L.W., HERBERMAN, R.B., KLUG, T.L. & ZURAWSKI, V.R., JR. (1984). Initial clinical evaluation of an immunoradiometric assay for CA 19-9 using the NCI serum bank. *Int. J. Cancer,* **33,** 339.